# A study of the interplay effect for VMAT SBRT using a four‐axes motion phantom

**DOI:** 10.1002/acm2.12947

**Published:** 2020-06-23

**Authors:** Jermey Leste, Imene Medjahed, François‐Xavier Arnaud, Regis Ferrand, Xavier Franceries, Manuel Bardies, Luc Simon

**Affiliations:** ^1^ INSERM Toulouse France; ^2^ IUCT‐Oncopole Toulouse France

**Keywords:** interplay, motion, radiotherapy, respiratory, stereotactic body radiation therapy (SBRT), volumetric modulated arc therapy (VMAT)

## Abstract

**Purpose:**

To assess the accuracy of volumetric modulated arc therapy (VMAT) stereotactic body radiation therapy (SBRT) when treating moving targets (such as lung or liver lesions), focusing on the impact of the interplay effect in the event of complex breathing motion and when a gating window is used.

**Methods:**

A dedicated programmable motion platform was implemented. This platform can carry large quality assurance (QA) phantoms and achieve complex three‐dimensional (3D) motion. Volumetric modulated arc therapy SBRT plans were delivered with TrueBeam linac to this moving setup and the measured dose was compared to the computed one. Several parameters were assessed such as breathing period, dose rate, dose prescription, shape of the breathing pattern, the use of a planning target volume (PTV) margin, and the use of a gating window.

**Results:**

Loss of dose coverage (D95%) was acceptable in most situations. The doses received by 95% of the CTV, D95% ( $CTV_{m}$) ranged from 94 to 101% (mean 98%) and the doses received by 2% of the CTV D2% ( $CTV_{m}$) ranged from 94% to 110% of the prescribed dose. A visible interplay effect was observed when no margin was used or when the number of breathing cycles during the treatment delivery was lower than 20.

**Conclusions:**

In our clinical context, treating lung and liver lesions using VMAT SBRT is reasonable. The interplay effect was moderated and acceptable in all simulated situations.

## 1 INTRODUCTION

In 2018 an important review article showed that treatment outcomes for early stage non‐small cell lung cancer treatment using stereotactic body radiation therapy (SBRT) are comparable with surgery, especially for patients with medical comorbidities.^1^ Stereotactic body radiation therapy is routinely used and clinical outcomes at 10 yr have been reported.^2^ The debate is no longer about whether SBRT is beneficial to lung inoperable cancers but whether it is for operable ones.^3^ Encouraging results have also been reported for pancreatic cancer^4^ and for local tumor control of primary and secondary malignancies of the liver.^5^, ^6^, ^7^

Radiotherapy of these areas must take respiratory movement into account^8^ to avoid discrepancies between the expected and the delivered dose. Typically, the amplitude of this movement during free breathing (FB) ranges from 8 to 15 mm.^9^ Nevertheless, higher motion amplitudes, ranging from 30 to 40 mm, have been reported for the lung,^10^ the pancreas,^11^ and the liver.^12^

It is generally assumed that the motion is mainly in the superior–inferior (SI) direction and is greater for tumors located in the lower lobe. However, the breathing motion is highly patient dependent. A study reported a lung tumor motion for which the preponderant component was anterior–posterior (AP).^13^ Moreover, in another study of 11 patients, the most mobile lung tumor was not observed in the lower lobe but observed in the upper one.^14^

Several methodologies and technologies have been proposed to treat these moving clinical target volumes (CTV). These methods have been known for more than 20 yr and have not changed much during that time.^9^ Of the large families of methods, the two most used are deep‐inspiration breath hold^15^ (DIBH) and internal target volume (ITV) based methods. With ITV methods, the treatment can be delivered during either the complete breathing cycle (BC) or only during a fraction of it using an external surrogate (gated RT). The ITV is drawn according to the CTV motion, generally observed on a four‐dimensional computed tomography (4D‐CT).

The latter method is attractive for treating SBRT because beam durations are generally long and poorly compatible with a DIBH. However, when the ITV method is used, two effects can deteriorate the expected dose distribution: dose blurring and, if the fluence is modulated, the interplay effect (IE).

Dose blurring is the simple effect that deteriorates the dose distribution when the target is moving while the computation is made using a static CT.^16^ For very simple geometries and motion, this phenomenon is well predicted by a convolution of the static dose distribution with the BC pattern.^17^ Such a calculation is possible only if the environment surrounding the planning target volume (PTV) can be considered as static from the point of view of the beam (e.g., an arc delivered to a homogeneous cylinder that moves along its axis in the SI direction). Otherwise the calculation may be distorted because of geometry changes, for example, source skin distance.

The IE is the unfortunate combination of motion of both the CTV and the multi‐leaf collimator (MLC).^18^ This combination can lead to hot or cold spots in CTV or organs‐at‐risk (OAR) that are difficult to foreseen. The IE was reported to be more important when the number of fractions decreases,^19^ when the PTV margin decreases,^20^ when the number of arcs decreases and when the dose rate increases.^21^ Recently, significant changes were observed for a single fraction of FFF volumetric modulated arc therapy (VMAT) liver SBRT, with a reduction of $D_{98\%}$ in the CTV up to −5%.^22^

In our department to take into account the tumor motion a six‐phase 4D‐CT is used to create two ITV volumes: $ITV_{6}$, the union of CTV seen on all the six breathing phases of the 4D‐CT (0, 16, 33, 50, 66, and 83%), and $ITV_{3}$ created only with exhalation phases (33, 50, and 66%). $PTV_{6}$ and $PTV_{3}$ are created by adding a 5 mm margin to $ITV_{6}$ and $ITV_{3},$ respectively. So $PTV_{6}$ is the target volume for delivery during FB while $PTV_{3}$ is the target volume for delivery within a gating window centered on the exhalation phase.

The lung and liver tumors are treated during FB; but if the motion amplitude induces a difference in volume superior to 50% ( $PTV_{6}/PTV_{3}>1.5$), the treatment is administered using a phase gating window between 33% and 66% with an external marker (RGSC, Varian Medical Systems, Palo Alto, CA), planned on $PTV_{3}$. Otherwise, the patient is treated during the full breathing cycle (planned on $PTV_{6}$).

The aim of this study was to assess the importance of the IE for VMAT SBRT in this clinical context before starting to treat patients. For this purpose, an original motion platform was developed that can carry large quality assurance (QA) phantoms. Using this setup, the influence of a large number of treatment parameters were assessed, such as the motion period, the shape of the breathing pattern, the use of a CTV‐PTV margin, and the use of a gating window. Measured doses acquired during programmed motion were compared to planned doses.

## 2 MATERIALS AND METHODS

### 2.A Programmable motion platform and phantom

**Fig. 1:**
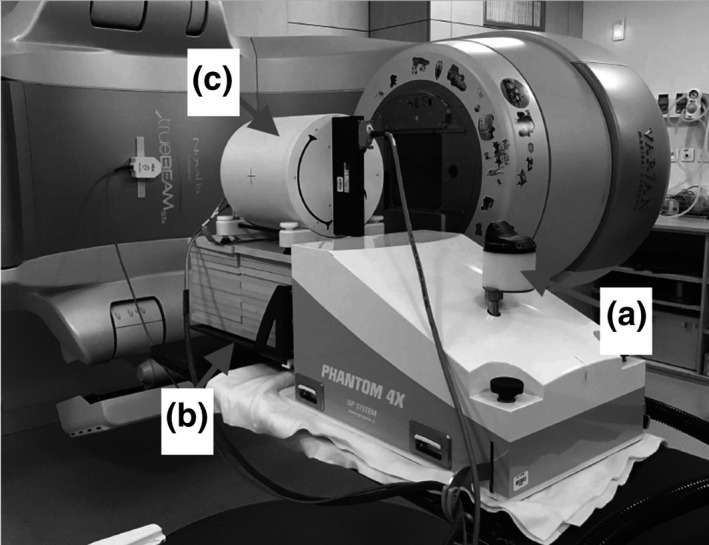
The programmable motion platform 4X has four independent and programmable motion axes. One axis (a) is dedicated to achieving a vertical motion for the secondary tray (for RGSC external marker). The three others are to perform x, y, and z motions for a main carbon tray (b). For example it can carry the PTW Octavius four‐dimensional phantom (c).

A programmable motion platform (PMP) with four independent motion axes was developed by our team in collaboration with a local manufacturer (PHANTOM 4X, ISP System, Vic en Bigorre, France). This PMP can simultaneously achieve three‐dimensional (3D) motion of a main tray for large QA phantoms and one‐dimensional (1D) motion (AP) of a small secondary tray for the RGSC marker (Fig. 1). Simple breathing pattern (e.g. sinusoidal curve) or complex motion can be programmed.

The PMP weighs 60 kg. The main tray is radio‐transparent and can support up to 35 kg. In this study, the fourth axis of the secondary tray (RGSC marker) always had the same amplitude (20 mm). Moreover the periods of the four axes were set to the same value.

### 2.B Programmed motion

Different 1D, two‐dimensional (2D), and 3D motions were programmed for the main platform by changing the following parameters: amplitude, period, breathing pattern. The list of these motions described by a *letter‐ID* is shown in Table 1.

**Table 1:** List of programmed motions. LR, SI, and AP: peak‐to‐peak amplitude (mm) for left‐right, anterior–posterior, and superior–inferior components, respectively.

ID	LR	SI	AP	Shape
A	0	10	0	*SIN*
B	0	20	0	*SIN*
C	0	25	0	*SIN*
D	0	25	20	*SIN*
E	0	25	20	*CUR*
F	15	25	20	*SIN*
G	15	25	20	*CUR*

#### 2.B.1 Breathing pattern

Two different breathing patterns were used for a given axis: simple sinus (denoted *SIN*) and a previously published^23^ model (denoted *CUR*). The *SIN* motion along a direction x is described by the Eq. (1):

(1) $$x=\frac{1}{2}A\cdot sin\left(\frac{2\pi \cdot t}{T}\right)$$

where t, A, and T are time, peak‐to‐peak amplitude, and the period, respectively.

**Fig. 2:**
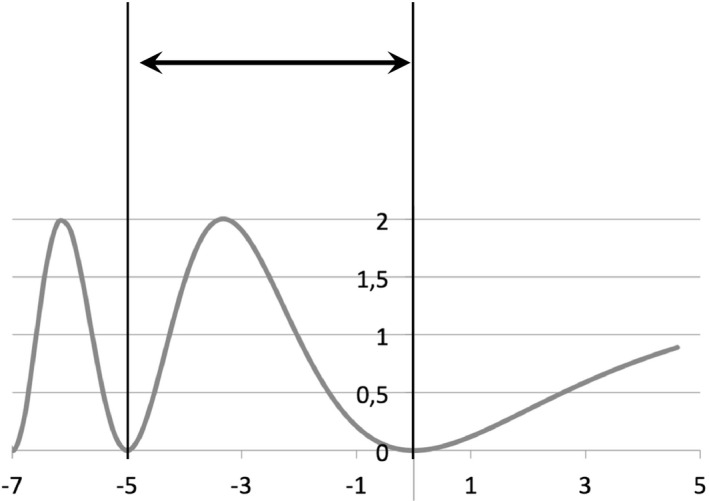
Breathing pattern according to the *CURIE* model: the part between vertical lines of this curve [see Eq. (2)] is repeated as a breathing pattern.

The CUR motion is obtained by creating a loop, using the part between ‐T and 0 of Eq. (2) (see Fig. 2).

(2) $$x=A\cdot \sin^{2}\left(e^{-\ln\left(\pi +1\right)\cdot \frac{t}{T}}-1\right)$$

Phase shifts are introduced for 2D and 3D motions to create cyclic trajectories.

#### 2.B.2 Period

All motions in Table 1 were programmed with a period of 5 s. To study the effect of the period, motions A, B, and C were also programmed with 3 and 7 s periods.

### 2.C SBRT planning

Using Eclipse v13.7 (Varian Medical Systems), VMAT SBRT plans were achieved, based on the synthetic images of the Octavius 4D (PTW, Freiburg, Germany) phantom: a cylinder with homogeneous density (0 HU) according to the methodology recommended by the manufacturer. Each plan was made with only one arc (from 90 to 270), collimator angle was set to 0. The tongue and groove effect is not taken into account in this study. The dose prescription was normalized to the median of the target volume.

A CTV was chosen as a sphere located at the center of the phantom (diameter 2 cm). Moreover three virtual OARs were created to ensure a large modulation of the VMAT plans. These OARs were arranged asymmetrically so that the solicitation of the leaves was different depending on their position in the MLC. The initial position of the CTV sphere was chosen to be the mid‐position of the motions. No 4D‐CT was acquired for this study. $ITV_{6}$ and $ITV_{3}$ were drawn by determining the position of the CTV during the different chosen motions. $ITV_{6}$ and $ITV_{3}$ correspond to the union of the CTV during the full BC (six phases) and during exhalation only, respectively (three phases, 33%, 50%, and 66%). $PTV_{6}$ and $PTV_{3}$ were created by adding a 5 mm margin to $ITV_{6}$ and $ITV_{3},$ respectively. Thus, for each motion of Table 1, four plans were created: with or without margin (targeting on PTV and ITV, respectively) and with or without gating (planning on $PTV_{3}$/ $ITV_{3}$ or on $PTV_{6}$/ $ITV_{6}$, respectively).

All plans were delivered using a TrueBeam linac (Varian Medical Systems) with 6 MV FFF fields (free flattening filter) at the maximum available dose rate (1400 MU/min) and HD120 MLC. To study the influence of dose rate, supplementary plans were created with 6 MV FF fields (600 MU/min dose rate) for motions A, B, and C (period 3, 5, and 7 s). Moreover, prescribed dose was 11 Gy for all plans but supplementary plans were made with a dose of 4 Gy (motion C) to assess the influence of prescribed dose.

The dose computation is performed with AAA algorithm (using a 0.25 cm grid). To be considered valid, plans had to meet the following arbitrary criteria: D95%(PTV)>95% and Dmax(PTV)<106%.

The following formalism is used hereafter: $N_{m,g}$ refers to an acquisition where N is the ID of the motion (see Table 1), m is the ITV‐PTV margin (0 or 5 mm), and g refers to the use or not of gating (y or n). For example, $C_{5,y}$ refers to an acquisition achieved using respiratory gating, during motion C, for a plan prepared using a PTV drawn by adding a 5 mm margin to the sum of CTVs at the three exhalation phases.

### 2.D Data acquisition

An ionization chamber array dedicated to SBRT (1000 SRS, PTW) was inserted in the phantom (Octavius 4D), which is placed on the PMP main tray. This phantom is used for our clinical routine for the QA of SBRT patients (global gamma index 2% — 2 mm). The array was always perpendicular to the beam axis because the phantom followed the rotation of the gantry thanks to an inclinometer. Thus, the PTW acquisition software (Mephysto) reconstructed a 3D dose matrix in a homogeneous water‐equivalent cylinder.^25^ To center the phantom (Octavius 4D) at isocenter, a PMP *stop position* was first programmed. The external marker (RGSC) was positioned on the dedicated secondary tray to ensure the possibility of a 33‐66% gated treatment. During the delivery, the PMP was moving with the motion corresponding to the chosen plan. Acquisition was started at a random phase of the BC.

### 2.E Data analysis

The acquired 3D dose matrices were recorded and CTV dose volume histograms (DVH) were computed using an open source software for medical image processing: 3D Slicer software (*slicer.org*). The doses received by 2% (D2%) and 95% (D95%) of the volume were recorded. These measured doses indexes in CTV (denoted $CTV_{m}$) were compared to the computed doses in PTV (denoted $PTV_{c}$), or ITV, if margin is zero, to check whether the clinical goal was achieved, for example, if the ITV method was effective (whatever the different effects). Moreover, to distinguish blurring (due to the motion) and the IE (due to the modulation and motion), measured dose profiles were compared to *blurred computed profiles*. Dose profiles were plotted in the SI direction through the center of the CTV. As previously explained, *blurred computed profiles* cannot be obtained by a simple convolution (as described in a recent publication^17^). Thus, these profiles were obtained using the following method. Knowing the motion, the position of the phantom at the six phases was determined. Then, using the TPS, the beam isocenter was placed successively at these six positions, the dose was calculated and the SI profile was extracted. The summation of these six dose profiles was divided by six and compared to the measured ones. A comparison of the profiles was achieved using 1D gamma index pass rate: percentage of points with γ<1 (global, 2% – 2 mm, threshold 10% of maximum dose).

## 3 RESULTS

### 3.A Dose statistics

For all the measurements, the doses received by 95% of the CTV, D95% ( $CTV_{m}$) ranged from 94 to 101% (mean 98%) and the doses received by 2% of the CTV, D2% ( $CTV_{m}$) ranged from 94 to 110% (mean 102%). Table 2 reports the variations in dose coverage for all measurements as the difference between D95% ( $CTV_{m}$) and D95% ( $PTV_{c}$) (in %). Table 3 reports the variation in near‐maximum doses for all measurements as the difference between D2% ( $CTV_{m}$) and D2% ( $PTV_{c}$) (in %).

**Table 2:** Difference (in %) between D95% ( $CTV_{m}$) (measured data) and D95% ( $PTV_{c}$) (extracted from TPS). For example, −3.0% indicates that the measured D95% ( $CTV_{m}$) is 3% smaller than the planned value D95% ( $PTV_{c}$), which reflects an under‐dosage during delivery. Variations greater than 3% are in bold. M.: motion pattern (see Table 2), Px: motion period (in s).

E	M.	Dose	CTV‐PTV 0 mm						CTV‐PTV 5 mm					
			No gating			Gating			No gating			Gating		
			P3	P5	P7	P3	P5	P7	P3	P5	P7	P3	P5	P7
3*FF	A	3*11 Gy							2.1	2.0	1.9			
	B								2.1	2.0	1.7			
	C			0.5			1.6		2.0	1.8	2.3		2.6	
8*FFF	A	7*11 Gy		2.1			1.8			**3.5**			1.4	
	B		**−3.6**	**−3.9**	**−4.5**	**−4.0**	**−4.0**	**−3.9**	−2.0	−2.0	−2.2	−0.3	−0.6	−0.5
	C			0.6	1.0		1.4	1.6		1.7	0.7		2.1	2.1
	D			1.6			0.5			**3.9**			**3.3**	
	E			1.8			−1.0			1.9			1.9	
	F			1.2			0.6			**4.2**			**3.5**	
	G			2.7			1.7			**4.9**			**4.8**	
	C	4 Gy		‐1.1			0.3							

**Table 3:** Difference (in %) between D2% ( $CTV_{m}$) (measured data) and D2% ( $PTV_{c}$) (extracted from TPS). Variations greater than 3% are in bold. M.: motion pattern (see Table 2), Px: motion period (in s).

E	M.	Dose	CTV‐PTV 0 mm						CTV‐PTV 5 mm					
			No gating			Gating			No gating			Gating		
			P3	P5	P7	P3	P5	P7	P3	P5	P7	P3	P5	P7
3*FF	A	3*11 Gy							0.3	0.0	0.2			
	B								**4.4**	3.0	0.4			
	C			2.1			**4.9**		**4.5**	1.2	0.9		1.8	
8*FFF	A	7*11 Gy		2.4			2.5			1.4			2.1	
	B		**4.2**	−2.0	−1.5	−1.0	−0.9	−0.8	−1.5	−1.7	−2.4	−2.3	−2.3	−2.2
	C			2.0	1.6		**4.8**	1.6		−0.4	−0.4		0.1	0.1
	D			0.5			0.0			**−8.8**			−1.6	
	E			0.8			0.5			0.2			0.4	
	F			−0.1			−0.1			−0.5			0.1	
	G			0.0			0.5			0.1			0.1	
	C	4 Gy		**8.6**			2.1							

### 3.B Dose profiles

**Fig. 3:**
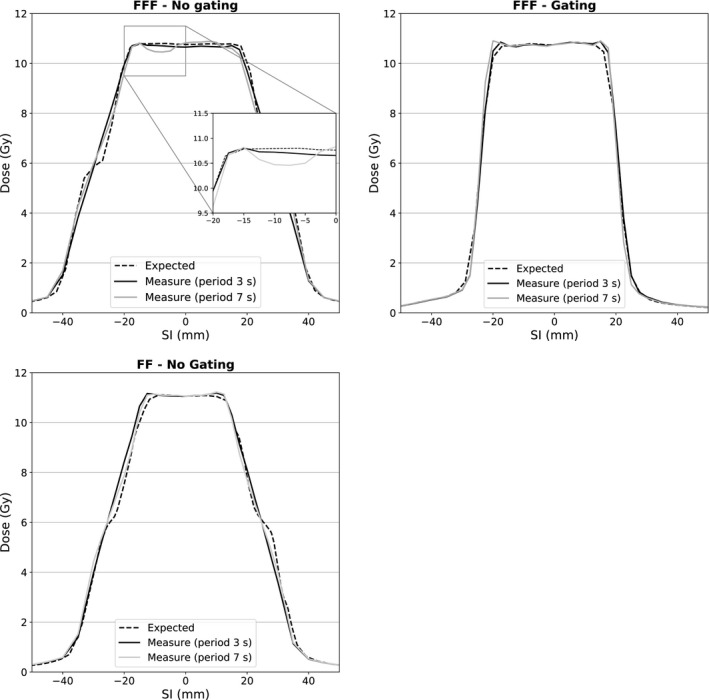
Superior–Inferior dose profiles passing through the planning target volume center for $B_{5,n}$ (up‐left), $B_{5,y}$ (up‐right) with 6FFF fields and $B_{5,n}$ with 6FF field (bottom‐left).

**Fig. 4:**
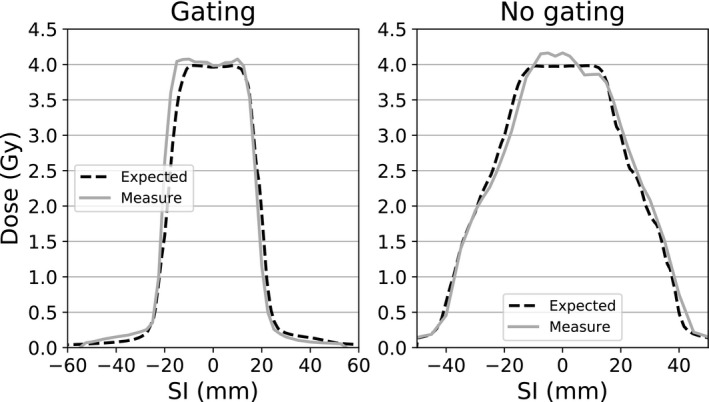
Superior–Inferior dose profiles passing through the planning target volume center for different plans with a prescription of 4 Gy with (left) and without (right) gating.

**Fig. 5:**
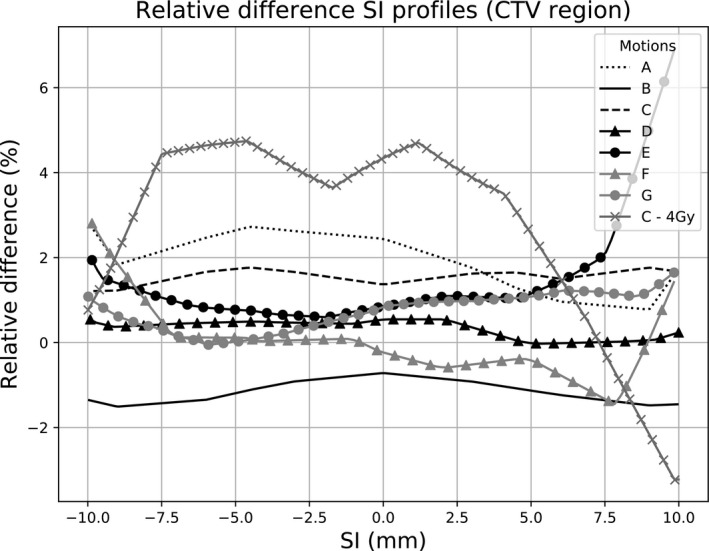
Relative difference (in %) within the clinical target volumes (CTV) region between computed and measured SI profiles. All curves are for motion period of 5 s and without gating. The first seven curves (a–g) are for a prescription of 11 Gy with a CTV‐PTV margin of 5 mm and the last one (C‐4 Gy) is for only 4 Gy with no margin, see Fig. 3).

In addition to the tables, Figs. 3 and 4 show examples of profiles to illustrate the IE. All these profiles are plotted through the center of the CTV in the SI direction. A comparison of measured and expected *blurred computed* dose profiles within the CTV region is reported in Fig. 5. Table 4 reports the GIPR (2%–2 mm) of the profiles plotted in Fig. 5.

**Table 4:** GIPR ( γ<1) for 1D global gamma index 2%–2 mm. Comparison of measured profiles to computed profiles.

ID	1D—GIPR (%)
A	98.0
B	100.0
C	100.0
D	94.5
E	94.1
F	100.0
G	86.2
C‐4 Gy	87.6

## 4 DISCUSSION

To study the IE during VMAT SBRT, a dedicated PMP was implemented. This PMP allowed us to move large QA phantoms and to simulate simple and complex breathing motions. Dynamic phantoms have already been used to test image quality and dose delivery,^26^ study the tumor tracking^27^ or the IE^17^ (only for 1D motion). To our knowledge, this is the first evaluation of blurring and interplay during VMAT SBRT delivered to an object animated with a 3D motion. It is also the first study of these effects when an exhalation gating window is used.

The purpose of this work was to assess the importance of these difficultly foreseeable effects in a clinical context. The first issue was simply to check whether the dose distribution really delivered to the CTV is different from the computed PTV dose presented to the radiation oncologist during planning. This assessment was mandatory before starting to treat patients in order to avoid dose discrepancies and especially cold spots.

The second issue was to separate blurring and the IE in order to assess the relative importance of each one. As the convolution approach used by Edvardsson et al.^17^ was not usable in our context of a more than 1D motion, another method was implemented, based on the accumulation of the dose computation at different motion phases.

Different 1D, 2D, and 3D motion patterns were implemented: simple sinus curves and also more complicated models. The *CUR* model is a pattern that reflects both the exhalation/inhalation duration ratio (i.e., 2/1) and the speed difference between inhalation and exhalation (exhalation speed is lower).^23^ These issues are not modeled by the $sin^{4}$ or $sin^{6}$ curves that are usually used.^25^

The magnitude of the variation in D95% is moderate (range: −4.5% to 4.9%) which means that the dose delivered to the CTV is sufficient for all configurations (see Table 2). In general, there is no loss of dose coverage (negative values in Table 2). The variation in D2% ranged from −8.8% to 8.6%, and is inferior to 3% for 47 cases on 54. A systematic decrease of D2% and loss of dose coverage is observed for the motion B.

The influence of several parameters on dose distribution is shown in Tables 2 and 3. First, the influence of the breathing period was very slight. For example, the loss of D95% for $B_{5,n}$ was −2.0%, −2.0%, and −2.2% for a motion period of 3, 5, and 7 s, respectively. Nevertheless, the period and the dose rate (between FF and FFF fields) modify the total number of BCs during delivery. Figure 3 shows that there is no visible IE on the dose profiles, except when the number of BC decreases to approximately n=20 (for a FFF dose rate of $1400MU.min^{-1}$ and a period of 7 s). This beneficial effect of low dose rate on the IE was already observed. Ong et al reported a strong limitation of IE when using FF fields instead of FFF.^21^ However, even when using FFF fields and during slow breathing motion, the CTV received a sufficient dose and D95% was greater than 99.3% in this configuration.

The other important issue was to assess the benefit of the ITV‐PTV margin. From a strictly mathematical point of view, with or without any ITV‐PTV margin, blurring should not be observed in the CTVs of this study. Indeed, ITVs were designed with a complete knowledge of the motions, and these motions were perfectly regular and reproducible. However, the only unacceptable cases were those for which no ITV‐PTV margin was used. For these cases, we observed cold spot in the CTV (D95% < 95%) and also large differences between planning and delivery (i.e., for Motion B, with or without gating, see Table 2).

Moreover the results showed that adding a margin tends to increase the dose coverage (mean variation in D95%: +0.1% without margin and +1.8% with margin) and reduce hot dose spot within the CTV (mean variation in D2%: +1.3% without margin and −0.01% with margin).

While it may not be immediately evident why the IE is greater when no margin is used around the ITV, it should be remembered that IE is created by the shadow of the MLC leaves on the CTV. When the MLC is delivering a modulated fluence, the probability of hiding a part of the ITV is smaller, when the ITV‐PTV margin increases. In other words, for a moderate leaf motion, the ITV is not hidden if the margin is wide enough. This issue was already reported by Li *et al.* Using a computed model, they observed a loss of mean PTV dose of 16.6% and less than 1% to the CTV.^20^

In our department, we use an exhalation gating window, when the tumor motion is great. Compared to a treatment during the full BC, the IE should be lower when this window is used. Indeed, when gating is used, the residual motion is limited to a few millimeters during beam ON periods. Actually, this influence is moderate for cold spots and the values in Table 2 with or without gating are very similar. Mean variation in D95% for all configurations are 0.9% and 0.8% without and with gating, respectively. There are also no important differences in D2% (see Table 3), mean variation in D2% is 0.7% without gating and 0.5% with gating window. Although a greater IE was visible on the SI profiles of Fig. 3 when gating was not used ( $B_{5,n}$ vs. $B_{5,y)}$), these differences are not confirmed by a difference of D95% or D2%. Reducing the IE is not the main reason for using a gating window. The main benefit of doing so is to reduce the PTV and hence the dose to the OARs. In this study, the PTV was reduced by 50% when ITV was drawn only on exhalation phases (from 33% to 66%).

A large variety of breathing pattern was tested, changing the amplitude, the shape, and from 1D to 3D, setting the other parameters to realistic clinical conditions (period 5 s, prescription 11 Gy, without gating). The impact on the interplay can be visually appreciated on Fig. 5. Moreover Table 4 shows quantitative comparisons (GIPR). All values are superior to 95% except the last two values. Excepting these values the 1D motions seems to have a lower IE (mean 1D GIPR 99.3%, mean 2D‐3D GIPR 93.7%).

The worse profile (excluding the one with a prescription of 4 Gy discussed hereafter) is for a 3D CUR pattern (profile G, GIPR 86.2%). This is probably because motion G had parallel components (3D motion) to displacement of the MLC leaves. Moreover the inhalation phase of this motion is shorter than the SIN pattern. Even for this motion, the CTV dose statistics were clinically acceptable.

The variation induced only by the IE on the SI profiles within the CTV region are mainly within 3%. We can notice a hot dose spot for motion E which reaches +7% and that there is a decrease of the CTV dose along the entire SI profile for motion B. These effects are attributed to the IE only that has a random component. However it is difficult to deduce a clear impact of the breathing pattern and as a general result, it can be stated that it had a poor influence on the IE.

Finally the key parameter of the IE was the number of BC during the delivery. We already discussed the importance of period and dose rate. It can also be seen in Fig. 4 that when the dose prescription was lower and thus the delivery time was reduced (11 Gy ≈ 2.5 min, 4 Gy ≈ 1 min), the IE was greatly increased (Fig. 4). GIPR was only 87.6% (Table 4) for this SI profile and differences within the CTV region are more important: from −3.3% to +4.7% (Fig. 5).

In that case, the use of gating greatly reduced this effect. However, a single fraction of 4 Gy is not clinically relevant and such a fractionation would be achieved with a number of fractions >5. The number of fractions is known to limit the IE by averaging it.^17^, ^19^ Increasing the number of fractions can also be interpreted as an increase in the number of BCs during the delivery.

In our clinical conditions (FFF fields, dose > 10 Gy with the use of a 5 mm CTV‐PTV margin and a gating window when necessary), the IE was acceptable. On the basis of this study, we now plan to move forward from a dynamic conformal arc technique to VMAT for the treatment of lung and liver using SBRT.

## 5 CONCLUSION

We have developed a four‐axes motion platform that is able to carry large QA phantoms to assess the IE during VMAT SBRT for moving targets. Several parameters were studied such as the amplitude, the period, the shape and the number of dimensions of the breathing pattern, as well as the use of a gating window or CTV‐PTV margin. IE was generally moderated and the only parameter that created a significant dose change in the CTV was the number of breathing cycles during the delivery. When this number was greater than 20, no important IE was observed.

## CONFLICT OF INTEREST

None.
